# Urethral Leiomyoma: A Rare Clinical Entity

**DOI:** 10.1155/2016/6037104

**Published:** 2016-11-23

**Authors:** Ng Beng Kwang, Aruku Naidu, Azyani Yahaya, Lim Pei Shan

**Affiliations:** ^1^Department of Obstetrics & Gynaecology, UKM Medical Centre, Kuala Lumpur, Malaysia; ^2^Department of Obstetric & Gynaecology, Hospital Raja Permaisuri Bainun, Ipoh, Malaysia; ^3^Department of Pathology, UKM Medical Centre, Kuala Lumpur, Malaysia

## Abstract

Extrauterine leiomyomas are encountered occasionally, which can pose a diagnostic dilemma and challenge to the gynaecologist. We report a rare case of urethral leiomyoma. A 31-year-old woman with history of primary subfertility presented with mass at her urethral meatus and lower urinary tract symptoms. She underwent examination under anaesthesia and excision of the urethral mass. Histopathological examination confirmed leiomyoma. Diagnosis and management of this common growth situated at a rare location were reviewed and discussed.

## 1. Introduction

Leiomyomas are benign tumours of smooth muscle, which commonly occurred at the genitourinary tract like uterus. Extrauterine leiomyoma are rare especially in the deep soft tissue like female urethra. Thus, presence of a urethra mass poses a significant diagnostic challenge as its differential diagnosis includes an extensive list of both benign and malignant pathologies of gynaecological or urologic origin. We describe an unusual case of urethral leiomyoma that was mistaken as urethral prolapse.

## 2. Case Report

**Figure 1:**
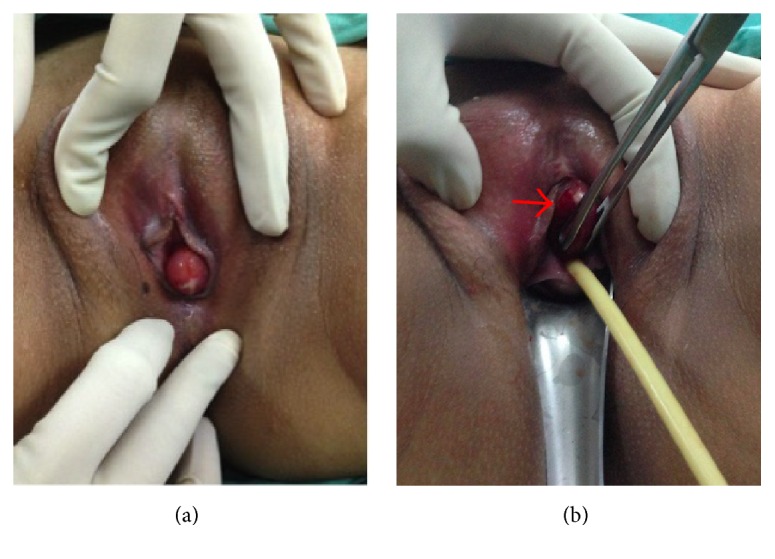
(a) A firm mass measuring 2.0 × 1.5 × 1.5 cm on the urethral meatus and (b) its relationship with the urethral meatus (arrow indicates the urethral mass).

A 31-year-old nulliparous with primary subfertility for 4 years presented with one-week history of painful swelling at the urethral meatus associated with lower urinary tract symptoms. She has dysuria and incomplete voiding but no haematuria. There was no significant medical or surgical history. Physical examination revealed a firm mass measuring 2.0 × 1.5 × 1.5 cm at the ventral wall of urethral meatus from 10 to 2 o'clock (Figure 1). Following an initial diagnosis of urethral prolapse, she was counseled for examination under anaesthesia and excision of the prolapsed urethra (Kelly-Burnham procedure). Intraoperatively, the patient was placed in lithotomy position. A 16 F Foley catheter was inserted. Haemostatic solution was prepared: bupivacaine hydrochloride 5 mg and adrenaline 5 mcg diluted 1 : 4 with water. Three millilitres of the solution was injected over the vaginal submucosa. Three stay sutures were placed at the base of the prolapse to mark the urethra mucosa. Prolapse was carefully trimmed off. The incision was sutured by simple interrupted method using absorbable 3.0 (mucosa-mucosa anastomosis).

**Figure 2:**
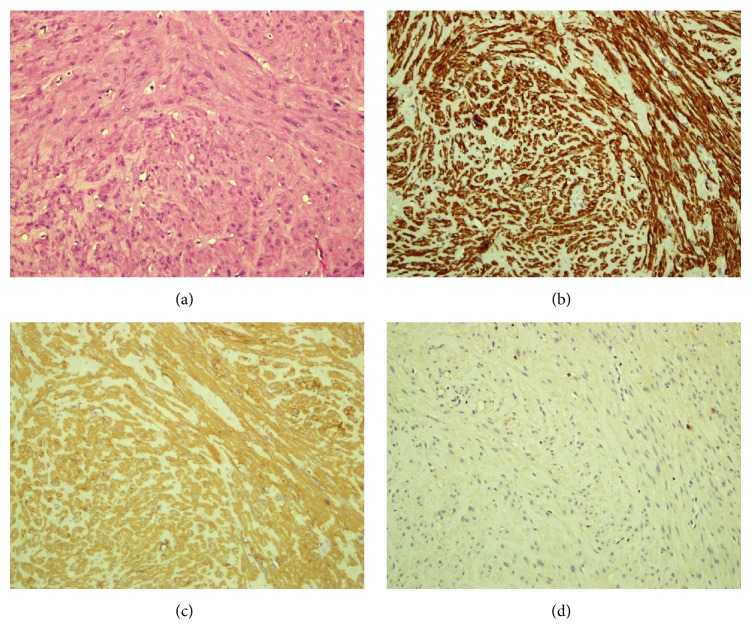
(a) HE 200x. Section shows a well-circumscribed tumour comprising interlacing bundles of smooth muscle fibres displaying uniform spindle-shaped nuclei. Immunohistochemical study shows the cells are positive for SMA (b) and desmin (c). They are negative for S-100 (d).

Histopathological examination (HPE) revealed a firm well-circumscribed mass measuring 20 × 15 × 14 mm. Microscopically, section showed a well-circumscribed tumour lined by stratified squamous epithelium and focal area of urothelial lining. The tumour was comprised of interlacing bundles of smooth muscle fibres displaying uniform spindle-shaped nuclei. There was no mitosis or nuclei atypia seen. Immunohistochemistry study showed that the cells were positive for SMA and desmin. The features confirmed diagnosis of urethral leiomyoma (Figure 2).

Postoperatively, she recovered well. The indwelling catheter was kept for 10 days and she was able to void without difficulty upon catheter removal. There was no recurrence observed during follow-up.

## 3. Discussion

Urethral masses are rare entity. The differential diagnoses include urethral prolapse, urethral caruncle, urethral diverticulum, Skene's duct cyst, Gartner's duct cyst, urethral carcinoma, and ectopic ureteroceles [1]. Urethral leiomyoma, found in this case, is exceptionally rare. Being classified as deep tissue leiomyomas, it is important to distinguish them from their malignant counterpart, leiomyosarcomas, which are more commonly found in deep soft tissue.

Urethral leiomyomas have a female preponderance. The tumour often found in women at reproductive age in their third and forth decades of life [2]. Moreover, the size of tumour decreased after menopause [3], which has led to speculation that the growth of urethral leiomyoma was hormonal dependent [2]. The tumour size was 1–7 cm on average [4–9]. This tumour commonly affects the proximal urethra and arises from the posterior wall of the urethra [4]. Diverse manifestation was observed depending on the size and location of the tumour. Patients might be presented with palpable mass [2, 4], haematuria [5, 9], acute urinary retention [6, 10], urinary tract infection [7], or vaginal bleeding [8]. However, up to 23% patients remained asymptomatic and underwent surgery incidentally [10]. This patient presented with a painful mass located at the distal urethra, which is relatively unusual.

A thorough assessment includes detailed history and physical examination is of utmost importance in the evaluation of a urethral mass. In selected cases, radiological imaging such as ultrasound, pelvic magnetic resonance imaging (MRI), or computed tomography (CT) scan might be helpful. Transperineal ultrasound showed well-defined iso- to hypoechoic homogenous mass with whorled appearance and significant internal vascularity on colour flow Doppler [11] whereas MRI revealed homogenous well-encapsulated mass appearing isointense on T1 and intermediate signal on T2 weighted image with intense contrast enhancement [11]. Imaging modalities may help to determine the exact location of tumour, depth of tissue infiltration, tissue plane, presence of features suggestive of malignancy, and finally the planning of surgical excision [12].

Due to the rarity of this condition, its management and treatment have not been established yet. Though local excision is usually recommended, hormonal treatment with gonadotrophin releasing hormone analogue had been described [13]. Complete surgical excision is usually achieved with very low risk of recurrence. So far, two cases of recurrence had been reported in the literature [14, 15]. Merrell and Brown reported a case of recurrent urethral leiomyoma that required three operations in 10 years [14]. The positivity for smooth muscle actin and desmin immunohistochemistry confirmed the muscular origin of the tumour cells and there were no features of malignancy to suggest leiomyosarcoma. [16]. Fortunately, to date, there had been no malignant transformation being reported.

## 4. Conclusion

Urethral leiomyoma is rare and poses diagnostic difficulty to a certain degree. Complete surgical excision was recommended for HPE confirmation.
